# A high-throughput screening method for evolving a demethylase enzyme with improved and new functionalities

**DOI:** 10.1093/nar/gkaa1213

**Published:** 2020-12-18

**Authors:** Yuru Wang, Christopher D Katanski, Christopher Watkins, Jessica N Pan, Qing Dai, Zhuoxun Jiang, Tao Pan

**Affiliations:** Department of Biochemistry and Molecular Biology, USA; Department of Chemistry, University of Chicago, Chicago, IL 60637, USA; Department of Biochemistry and Molecular Biology, USA; Department of Biochemistry and Molecular Biology, USA; Department of Biochemistry and Molecular Biology, USA; Department of Chemistry, University of Chicago, Chicago, IL 60637, USA; Department of Biochemistry and Molecular Biology, USA; Department of Biochemistry and Molecular Biology, USA

## Abstract

AlkB is a DNA/RNA repair enzyme that removes base alkylations such as *N^1^*-methyladenosine (m^1^A) or *N^3^*-methylcytosine (m^3^C) from DNA and RNA. The AlkB enzyme has been used as a critical tool to facilitate tRNA sequencing and identification of mRNA modifications. As a tool, AlkB mutants with better reactivity and new functionalities are highly desired; however, previous identification of such AlkB mutants was based on the classical approach of targeted mutagenesis. Here, we introduce a high-throughput screening method to evaluate libraries of AlkB variants for demethylation activity on RNA and DNA substrates. This method is based on a fluorogenic RNA aptamer with an internal modified RNA/DNA residue which can block reverse transcription or introduce mutations leading to loss of fluorescence inherent in the cDNA product. Demethylation by an AlkB variant eliminates the blockage or mutation thereby restores the fluorescence signals. We applied our screening method to sites D135 and R210 in the *Escherichia coli* AlkB protein and identified a variant with improved activity beyond a previously known hyperactive mutant toward *N^1^*-methylguanosine (m^1^G) in RNA. We also applied our method to O6-methylguanosine (O6mG) modified DNA substrates and identified candidate AlkB variants with demethylating activity. Our study provides a high-throughput screening method for *in vitro* evolution of any demethylase enzyme.

## INTRODUCTION

AlkB is an Fe(II)- and α-ketoglutarate (αKG)-dependent dioxygenase which catalyzes the oxidative demethylation of alkylated DNA and RNA and protects the bacterial genome against alkylation damages (1,2). It is well known to remove alkylation on nitrogen atoms of nucleobases, including methylations such as *N^1^*-methyladenosine (m^1^A), *N^3^*-methylcytosine (m^3^C), and bulkier alkylation damages such as 1, *N*^6^-ethenoadenine (εA) (1). Recently, it was discovered that the *Escherichia coli* AlkB and the human homologs ALKBH2 and ALKBH3 can also react with the carbon atom of 5-methylcytosine (5mC), oxidizing it to 5-hydroxymethylcytosine (5hmC), 5-formylcytosine (5fC) and 5-carboxylcytosine (5caC) (3). Among the nine mammalian homologs of AlkB, designated as ALKBH1–8 and FTO, only ALKBH2 and ALKBH3 have been confirmed to repair DNA *in vivo* while several others act on RNA substrates (4).

Transfer RNA (tRNA) is heavily chemically modified which hinders its direct sequencing as modifications frequently block the reverse transcription, reducing the formation of long cDNA products in sequencing. Recently, two studies utilized the *E. coli* AlkB enzyme to remove modifications from tRNA to facilitate efficient and quantitative tRNA sequencing (5,6). As a tool, AlkB was also used to facilitate the identification of m^1^A modification sites in mRNA by specifically eliminating mutation signatures caused by disruption to reverse transcriptase by m^1^A (7).

The preferred methylated substrates for the wild type *E. coli* AlkB are m^1^A and m^3^C; however, targeted AlkB mutants can have new substrate preferences (6,8–10). For examples, our laboratory has identified AlkB D135S mutant which has higher reactivity than wild type against N1-methylguanosine (m^1^G). AlkB mutant D135S/L118V is able to demethylate *N*^2^,*N*^2^-dimethylguanosine (m^2^_2_G) which is a poor substrate for the wild type enzyme (6,8). The Yi laboratory has found that the AlkB D135I mutant demethylates m^6^A in RNA (10). These results suggest that the AlkB protein could be engineered to accommodate non-endogenous substrates. AlkB mutants with improved or new activities are highly desirable, which can facilitate studies such as tRNA sequencing and identification of modifications in mRNA and tRNA. So far, AlkB engineering studies were performed by rational design based on crystal structures and low throughput screening of a limited number of variants (6,8–10). No studies have been conducted to systematically evaluate all variants at specific residues of AlkB due to a lack of a high-throughput evaluation platform.

Here, we introduce a Broccoli RNA-based fluorescence assay for high throughput screening of AlkB demethylation activity on RNA and DNA substrates. Using our assay, we systematically evaluated two functionally important residues in the *E. coli* AlkB protein, D135 and R210. Screening libraries with random mutations at position 135 for m^1^G demethylase activity, we recovered the hyperactive D135S mutant previously identified from targeted mutation, as well as new variants that exhibited improved activity compared to the wild type AlkB. Specifically, we identify D135T which is more active than D135S against m^1^G substrates. The D135T mutant is active against endogenous tRNA modifications for tRNA-seq experiments. In addition, we screened AlkB variants for a novel O6-methylguanosine (O6mG) demethylase activity on DNA. We identified positive hits which serve as a starting point for further engineering of AlkB.

## MATERIALS AND METHODS

### Materials

The 96-deep square well plate was purchased from Thomas scientific (1159Q92). Protease Inhibitor Cocktail powder for preparing *E. coli* crude lysate was purchased from Sigma Aldrich (P8465–5ML); Pierce™ Protease Inhibitor Mini Tablets EDTA free used for protein purification was purchased from Thermo Fisher Scientific (88666). SUPERase• In™ RNase Inhibitor (AM2696), lysozyme Solution (90082), DNase I (EN0521) and M-MLV RT (28025013) were purchased from Thermo Fisher Scientific. T7 RNA polymerase (M0251) and Taq DNA (M0273) polymerase were purchased from New England Biolabs Inc. ZR-96 Oligo Clean & Concentrator was purchased from Zymo Research (D4062). The 4x Laemmli sample buffer was purchased from Bio-Rad (161-0747). Nuclease P1 from Penicillium citrinum and shrimp alkaline phosphatase used for digesting yeast tRNA for LC–MS/MS analysis were purchased from Sigma Aldrich (N8630) and New England Biolabs Inc. (M0371), respectively.

### RNA and DNA oligonucleotides

Oligonucleotides for mutagenesis, reverse transcription, PCR and *in vitro* transcription and fluorescently labeled oligonucleotides for characterizing reverse transcription were purchased from Integrated DNA technologies (IDT) with standard desalt purification. The RNA oligonucleotides containing m^1^G and the DNA oligonucleotide containing O6mG were synthesized in-house on an Expedite DNA synthesizer using commercial m^1^G-phosphoramidite and O6-methyl-G phosphoramidite from Glen Research on 1 μmol scale with the same coupling times as the commercial unmodified phosphoramidites. Based on DMTr cation calculations, both modified phosphoramidites were coupled as efficiently as unmodified phosphoramidites. After synthesis, regular deprotection procedures recommended by Glen Research were followed. After deprotection, the RNA oligonucleotides were purified through HPLC with a C18 column and were eluted with 0–20% acetonitrile in 0.1 M triethylammonium acetate. The desired peak was collected and dried by lyophilization. Synthesized RNA and DNA were dissolved in 10 mM Tris–HCl, pH 7.5, and the quality was examined on a 15% PAGE-urea gel. All oligonucleotides looked pure on the gel and were used directly. Sequences of oligonucleotides used are listed in Supplementary Table S2.

### Preparation of AlkB variant plasmids

The *AlkB* D135S gene with deletion of amino (N) terminal 11 amino acids was cloned into a PET28a (+) vector to generate a 6x His-tag fusion construct. Dead mutant of AlkB was made following the QuikChange II XL Site-Directed Mutagenesis Kit (Agilent, 200521) using the AlkB D135S plasmid as the template. To make D135X and R210X libraries, saturation mutagenesis was introduced at positions 135 or 210 following the QuikChange II XL Site-Directed Mutagenesis Kit using primer oligonucleotides containing the degenerative codons NNS at the target positions and AlkB D135S as the template. The resulting mutagenesis products were transformed into XL 10 Gold *E. coli* cells following manufacturer's protocol. For each library, around 200 *E. coli* colonies were pooled to ensure coverage at least 10 times greater than the library diversity. The libraries were confirmed with Sanger sequencing.

### Testing AlkB protein expression in *E. coli* cells

*Escherichia coli* cells (2 × 10^7^) expressing AlkB plasmids were pelleted and supernatant was removed. The cell pellet was resuspended in 20 μl lysis buffer containing 10 mM Tris–HCl pH 7.4, 300 mM NaCl, 5% glycerol, 2 mM CaCl_2_ and10 mM MgCl_2_, boiled at 93°C for 3 min and then chilled on ice. Two microliter DNase I was added to each sample and the digestion was incubated at RT for 2 min. Two microliter of the digested sample was added with 15 μl lysis buffer and 6 μl 4× Laemmli sample buffer and then subjected to electrophoresis to visualize target proteins on a 4–12% Bis–Tris gel.

### Measuring RNase activity in *E. coli* crude lysate

RNase Activity Detection/Quantification Assay Kit (BioVision, cat# K934) was used to measure the RNase activity in *E. coli* crude lysate with and without the addition of RNase inhibitor. Following the manufacturer's protocol, 6 μl cell lysate and 0 or 3 μl SUPERase• In™ RNase Inhibitor were added in a standard 60 μl reaction. The fluorescence signals were recorded on a plate reader (BioTek, Inc.), with excitation wavelength of 472 nm and emission wavelength of 507 nm.

### Screening assay for RNA substrate

Plasmids encoding AlkB enzymes were transformed to T7 express competent *E. coli* cells (New England Biolabs) following the manufacturer's protocol. Single colonies were picked from culture plates and grown in 0.5 mL LB media supplemented with 50 μM kanamycin in a 96 deep square well plate (Thomas Scientific). Cultures were incubated at 37°C overnight with shaking at 250 rpm. The next day (cultures should all reach to the stationary phase), 80 μl of culture was used to inoculate 1.2 ml fresh media in a second 96 deep-square well plate and the culture was continued for 3–4 h at 37°C with shaking at 250 rpm (until OD_600_ was around 1). Subsequently, IPTG and freshly made FeSO_4_ solution were added to the culture to 1 mM and 5 μM, respectively, and the cultures were incubated for additional 4 h at 30°C with shaking at 250 rpm to induce AlkB protein expression from the plasmids. Cells were then pelleted to the bottom of plate wells by centrifugation at 4000 rpm for 30 min at room temperature. The pellets were each resuspended in 160 μL lysis buffer containing 10 mM Tris–HCl pH 7.4, 300 mM NaCl, 5% glycerol, 2 mM CaCl_2_, 10 mM MgCl_2_, 10 mM 2-mercaptoethanol, 1 mg/ml lysozyme and protease inhibitor (Sigma Aldrich) (5 μl was added per ml lysis buffer). Cell lysis was performed at 37°C for 1 h, with shaking at 250 rpm. Cell debris were pelleted from the lysate by centrifuging the plate at 4000 rpm for 30 min at 4°C and the supernatant was used for downstream experiments. The first overnight cultures were pelleted and saved in –80°C freezer for later usage as described below.

The demethylation reaction was conducted in a 50 μl mixture containing 25 mM MES buffer (pH 6.0), 2 mM MgCl_2_, 50 μM (NH_4_)_2_Fe(SO_4_)_2_•6H_2_O, 2 mM l-ascorbic acid, 300 μM α-ketoglutarate, 50 μg/ml BSA, 270 mM KCl, 1 U/μl SUPERase•In RNase inhibitor (Invitrogen), 4 pmol RNA with the m^1^G modification and 2 μl lysate supernatant. The reaction was incubated at 25°C for 1h and then quenched by adding EDTA to 2.5 mM and heating at 65°C for 10 min to denature the protein.

The product (2.4 μl) from the demethylation reaction was used directly in a reverse transcription reaction in a 20 μl scale containing 0.5 μM primer, 5 μM dNTP, 10 μM DTT, 2U/μl RNase inhibitor and 10 U/μl M-MLV RT in 1× RT buffer. The reverse transcription was performed following the manufacturer's protocol except that 5 μM dNTP was used. The reaction mixture containing the RNA template, the primer and dNTP was first heated at 65 °C for 5 min, followed by quick chill on ice. After the addition of the other components, the reaction was incubated at 37°C for 50 min, followed by denaturing the M-MLV RT by heating at 70°C for 15 min. Two μl of the reverse transcription product was used in a 20 μl scale PCR reaction and amplified using the Q5 high fidelity DNA polymerase (New England Biolabs Inc.) following the manufacturer's protocol. PCR followed the program of 98°C for 30 s; (98°C for 10 s, 60°C for 30 s, 72°C for 25 s) × 20 cycles; 72°C for 2 min and 4°C hold. The PCR products (7 μl) were then used in the *in vitro* transcription reaction (21 μl scale) containing 2 mM rNTP, 25 mM Mg^2+^, 10 mM DTT, 50 μM DFHBI-IT and 25 U T7 polymerase in 1× RNAPol buffer. The K^+^ ion needed for the Broccoli RNA G-quartet structure is supplied from the PCR reaction mixture at a final concentration of 17 mM. Soon after the T7 polymerase was added to the reaction, the fluorescence signal was recorded in a 384-well plate on a plate reader (BioTek, Inc.), with settings of 37°C incubation for the *in vitro* transcription reaction, excitation wavelength of 472 nm and emission wavelength of 507 nm. The *in vitro* transcription reaction was conducted for up to 3 h and fluorescence was recorded at an interval of one read per min.

Positive hits identified from the screening were tracked back to the first overnight *E. coli* culture based on corresponding locations in the 96-well plate. The genotypes of the AlkB mutants were determined by Sanger sequencing the *E. coli* cell cultures. For small-scale confirmation of the positive hits with the fluorescence assay, the same procedure as the screening assay was followed except that *E. coli* was grown and lysed in 1.5 ml eppendorf tubes.

### Screening assay for DNA substrate

The screening assay for the DNA substrate followed procedures for the RNA substrate described above with following modifications. In the demethylation reaction with DNA, 0.432 pmol DNA substrate was added in a 50 μl scale reaction, and the SUPERase•In RNase inhibitor was not used. Following the demethylation reaction, DNA substrates were purified with ZR-96 Oligo Clean & Concentrator with each sample eluted with 15 μl ddH_2_O. We have found that purifying DNA substrates after the demethylation reaction substantially improved the fluorescence signals in the assay. Seven μl of each sample was then used in a 15 μl scale PCR reaction with Taq DNA polymerase following the manufacturer's protocol. A nested PCR program was used: 95°C for 30 s; (95°C for 20 s, 51°C for 30 s, 68°C for 30 s) × 10 cycles; 68°C for 2 min; (95°C for 20 s, 60°C for 30 s, 68°C for 30 s) × 20 cycles; 68°C for 5 min; 4°C hold. The PCR products (7 μl) were then used in the *in vitro* transcription reaction while fluorescence was detected as described above.

### Purification of AlkB proteins

AlkB enzymes (wild type, D135S and S135T) were expressed in T7 express competent *E. coli* cells (New England Biolabs Inc.). Cells were grown in a 2 l scale LB medium at 37°C until the OD_600_ reached 0.4–0.8. Isopropyl β-d-1-thiogalactopyranoside (IPTG) and freshly made FeSO_4_ solution were added to the culture to 1 mM and 5 μM, respectively, and the culture was grown at 30°C for additional 4 h to induce the AlkB expression. Cells were pelleted and resuspended in lysis buffer containing 10 mM Tris–HCl pH 7.4, 300 mM NaCl, 5% glycerol, 2 mM CaCl_2_, 10 mM MgCl_2_, 2 mM 2-mercaptoethanol and 1× protease inhibitor (Thermo Fisher Scientific). Cells were then lysed with sonication and centrifuged at 13 000 rpm for 45 min. The supernatant was first purified with a manual Ni-NTA column and eluted with a buffer containing 10 mM Tris–HCl pH 7.4, 300 mM NaCl and 250 mM imidazole. The protein was then further purified using Mono S chromatography. The protein was eluted with gradient buffers composed of different ratios of buffer A (10 mM Tris–HCl pH 7.4 and 100 mM NaCl) and buffer B (10 mM Tris–HCl pH 7.4 and 1.5 M NaCl) and then buffer exchanged to the storage buffer (10 mM Tris–HCl pH 7.4, 250 mM NaCl). Glycerol was then added to 30%. The protein was aliquoted, snap frozen in liquid nitrogen and stored at –80°C.

### In vitro demethylation assay with purified proteins

Purified D135T or D135S (3.375 pmol) was mixed with 4 pmol m^1^G-broccoli RNA in a 50 μl mixture containing 25 mM MES buffer (pH 6.0), 2 mM MgCl_2_, 50 μM (NH_4_)_2_Fe(SO_4_)_2_•6H_2_O, 2 mM l-ascorbic acid, 300 μM α-ketoglutarate, 50 μg/ml BSA, 270 mM KCl, and 1 U/μl SUPERase•In RNase inhibitor (Invitrogen). In the control experiment, EDTA was added to 4 mM to chelate Fe^2+^ and Mg^2+^ ions required for the enzymatic reaction. The demethylation reaction was conducted at 25°C for 8 min and quenched by adding EDTA to 4 mM and heating at 65°C for 10 min. Then the demethylation product (2.4 μl) was reverse transcribed, followed by PCR and *in vitro* transcription as described above. The PCR was performed for 15 cycles.

A 9-mer RNA oligonucleotide (5′-GAGC(m^1^G)UUAG-3′) was used in the assay to measure enzyme kinetics for D135T and D135S mutants. Purified D135T or D135S protein (80 pmol) was mixed with various amounts of the oligonucleotide (40 pmol, 80 pmol, 120 pmol or 160 pmol) in a 20 μl mixture containing 25 mM MES (pH 6.0), 2 mM MgCl_2_, 50 μM (NH_4_)_2_Fe(SO_4_)_2_•6H_2_O, 2 mM l-ascorbic acid, 300 μM α-ketoglutarate, 50 μg/ml BSA, 270 mM KCl, and 1 U/μl SUPERase•In RNase inhibitor (Invitrogen). The demethylation reaction was conducted at 25°C for varying times and quenched by adding EDTA to 4 mM and heating at 65°C for 10 min. Demethylated RNA was purified by phenol–chloroform extraction and ethanol precipitation. The purified RNA was dissolved in 5 μl water and deionized through incubation with 20 μl cation exchange resin (BioRad, Cat #731–6214) for 20 min. The RNA was then analyzed by MALDI-TOF/TOF MS using 2′,4′,6′-Trihydroxyacetophenone (THAP) as matrix and negative ion reflector mode. The MALDI spectrum was analyzed using flexAnalysis software with areas of target peaks integrated to quantify the demethylation fraction. The resulting data was fitted to the equation *y* = A*(1 – exp(–k*t)), where *y* is the demethylation fraction at time *t*, *A* is the fitted reaction saturation point and *k* is the fitted rate constant (min^−1^). The values of *K*_M_, *k*_cat_ and *k*_cat_/*K*_M_ were obtained by fitting the data to the equation: ([S]/[E])*A*k = *k*_cat_[S]/(*K*_M_ + [S]), where [S] is the substrate concentration and [E] is the enzyme concentration.

In the demethylation assay with yeast tRNA (Sigma Aldrich, #10109517001), a modified protocol was used that contained no magnesium but still worked efficiently. Twenty pmol of purified D135T or D135S was mixed with 8 pmol (∼200 ng) yeast tRNA in a 25 μl mixture containing 50 mM MES pH 6.0, 2 mM l-ascorbic acid, 1 mM α-ketoglutarate, 0.3 mM (NH_4_)_2_Fe(SO_4_)_2_•6H_2_O, 0.1 M KCl, 50 ng/μl BSA and 4 U/μl SUPERase•In RNase inhibitor. In the control experiment, EDTA was added to 4 mM to chelate the Fe^2+^ ion required for the enzymatic reaction. The demethylation reaction was conducted at 37°C for 10 or 30 min and quenched by adding EDTA to 4 mM and heating at 65°C for 10 min.

In all demethylation reactions described in this paper, l-ascorbic acid, α-ketoglutarate and (NH_4_)_2_Fe(SO_4_)_2_•6H_2_O were made fresh right before usage.

### LC–MS/MS

Yeast tRNA (∼200 ng) from the demethylation reaction was purified using oligo clean & concentrator kit (Zymo Research). The tRNA was digested by nuclease P1 (1 U) in a 30 μl scale reaction containing 10 mM NH_4_HCO_3_ pH 5.3 at 42°C for 2 h, followed by digestion with shrimp alkaline phosphatase (0.5 U) in 1× CutSmart buffer (New England Biolabs Inc.) at 37°C for 2 h. The sample was diluted to 90 μl with RNase free water and filtered (0.22 mm pore size, 4 mm diameter, Millipore), and the solution was injected into LC–MS/MS. Nucleosides were separated by reverse phase ultra-performance liquid chromatography on a C18 column with on-line mass spectrometry detection using an Agilent 6410 QQQ triple-quadrupole LC mass spectrometer in positive electrospray ionization mode. The nucleosides were quantified by using the nucleoside to base ion mass transitions of 268 to 136 (A), 284 to 152 (G), 282 to 150 (m^1^A) with retention time of 0.8 min, 298.1–166.1 (m^1^G), and 258.2–126.1 (m^3^C). Quantification was performed in comparison with calibration curve obtained from pure nucleoside standards running on the same batch of samples. The ratios of m^1^G to G, m^1^A to A and m^3^C to G were calculated based on the calibrated concentrations.

### tRNA-seq data analysis

Paired-end reads (150 bp) from Illumina sequencing Hiseq 4000 platform were first merged using BBMap tools (Bushnell B. – sourceforge.net/projects/bbmap/) with the script ‘merge.sh’. Sequencing adaptors were trimmed off automatically during the step of merging reads. By design, reads contain 6-nt UMI sequences on the 5′ ends for labeling of unique sequences. Reads were first de-duplicated to remove PCR duplicates using the script ‘clumpify.sh’, followed by trimming the UMI sequences with the script ‘trim_UMI.sh’. Reads also contain sample-specific 3-nt internal barcode sequences on the 3′ ends which were introduced in early steps of the library constructing process to decrease the sample handling complexity. Based on the 3-nt barcode, data were decomplexed by searching sequences ending with the barcode sequences using the grep command with options of ‘-A 2 -B 1 ‘XXX$’’, where ‘XXX’ means the 3-nt barcode. After this, the 7-nt adaptor sequences (including the 3-nt barcodes) on the 3′ end of reads were further trimmed. The script used for these two steps is ‘sort_and_trim.sh’. The trimmed reads underwent quality control with options of ‘-t 20 -l 22′ using the script ‘quality_control_trim.sh’ to filter off reads with quality lower than 20 or length shorter than 22 nt. Genome was built using the script ‘build_genome.sh’ on the basis of the human tRNA genome sequence file named ‘hg19CCA+mito+spike-in.fasta’, which is modified from the hg19-mature-tRNA file downloaded from http://gtrnadb.ucsc.edu/Hsapi19/Hsapi19-seq.html, by removing identical genes, adding ‘CCA’ to the ends, and adding in the mitochondrial tRNAs. Reads were then mapped to the reference genome using bowtie2, and the resulting SAM files were converted to the BAM files, sorted and indexed using the script ‘map_hg19-CCA.sh’.

Base identities mapped to each position of the tRNA genome and read counts associated are analyzed from the sorted BAM files using the script ‘LS-bam-readcount.sh’, with the filter ‘-b 20’ to only count reads with a minimum quality of 20 at each nucleotide position. The resulting information were output as.txt files for further analysis. Then, mutations were called for each position from the .txt files using a python script ‘LC2_mut.py’ and the output information were saved as separate .csv files with names ending with ‘.mut.csv’ for further analysis. The information of mutation rates for the most abundant gene of each tRNA isodecoder were sorted out and data from different samples were combined, using an in-house R script ‘select_and_filter.R’. All scripts used in the analysis have been deposited in Github: https://github.com/yuruwang26/tRNA_seq_scripts.

## RESULTS

### Establishing a high-throughput screening platform for *in vitro* evolution of the AlkB enzyme

Broccoli RNA is a fluorogenic RNA aptamer which, upon folding to a tertiary structure, binds and induces the fluorescence of the fluorophore DFHBI-IT (11). Mutations at specific residues in conserved regions of this RNA can disrupt the RNA structure, thereby abolishing the fluorescence signal. Exploiting this property, Zhou *et al.* developed an assay that utilizes the Broccoli RNA containing an m^1^A modification as a platform to screen reverse transcriptase variants that can efficiently read through and cause mutation signatures at the site of modification (7). Inspired by this strategy, we adapted this approach to assay the demethylation activity of the DNA/RNA repair enzyme AlkB. In our strategy, an RNA oligonucleotide contains a reverse transcriptase (RT) stopping modification, such as m^1^G, which prevents synthesis of the full fluorogenic Broccoli RNA aptamer. Demethylation by an AlkB restores RT readthrough of the modified site and enables synthesis of the full-length Broccoli RNA aptamer, thus restoring the fluorescence signal.

**Figure 1.**
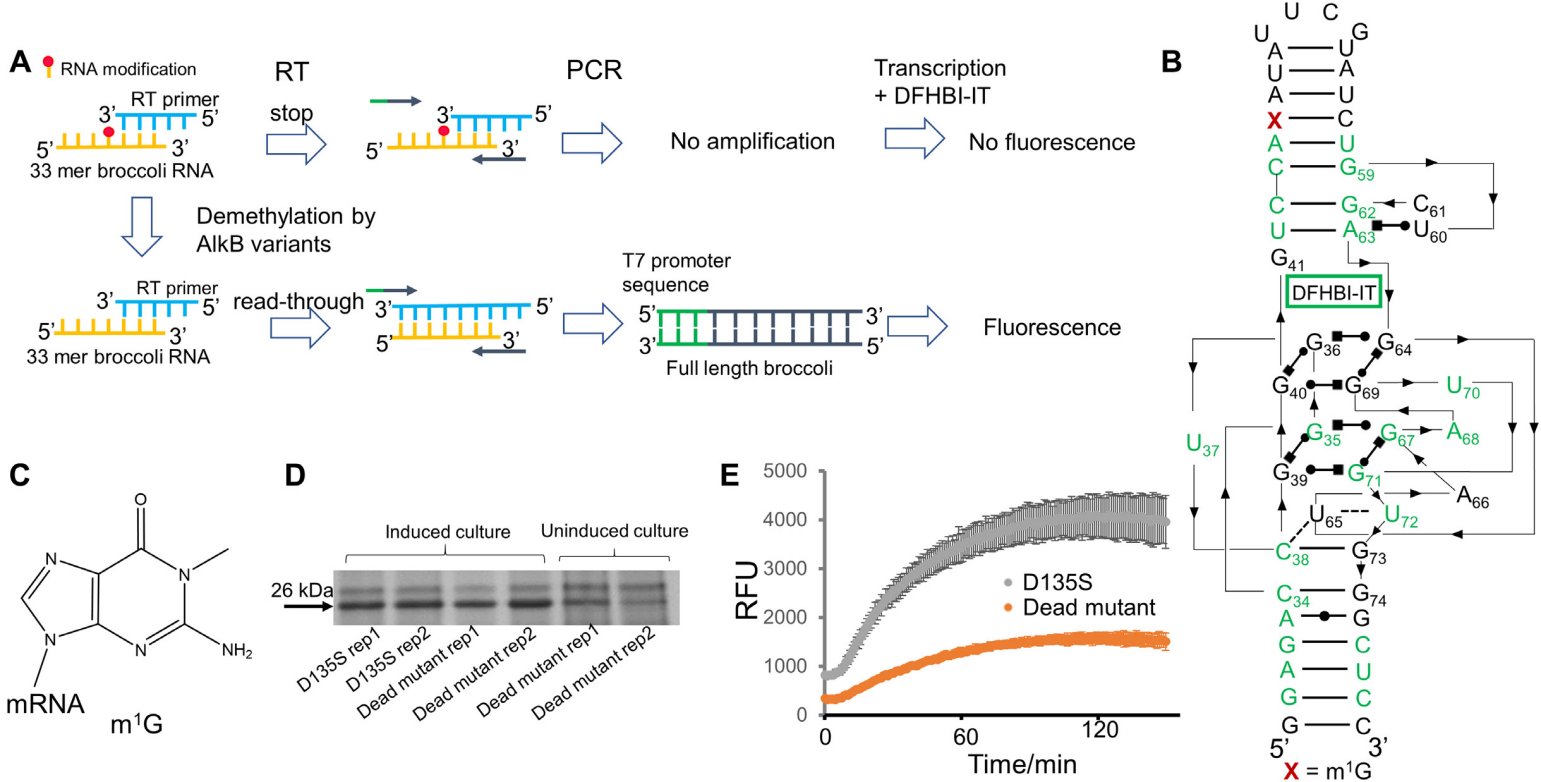
Establishment of the high-throughput screening system for AlkB. (**A**) The scheme of the Broccoli RNA fluorescence assay for high-throughput screening of AlkB activity towards modifications in RNA. RT: reverse transcription. DFHBI-IT is the fluorophore that the Broccoli RNA binds to. (**B**) The 51 nt Broccoli RNA sequence with conservative bases labeled in green. Thin lines with arrows denote chain connectivity and the Leontis-Westhof symbols denote canonical and non-canonical base pairs (26). The structure is based on the secondary structure of Spinach 1.2 and the high similarity of their core domains (12,13). The numbering scheme for Broccoli follows Filonov *et al.* (11) and is used throughout. The 51 nt consists of nucleotides numbering from 29 to 79, and the 33 mer substrate used in our assay consists of nucleotides numbering from 29 to 61, with m^1^G located at site 46. (**C**) The chemical structure of m^1^G. (**D**) D135S and the catalytically dead mutant are expressed at the same level in *E. coli* cells. (**E**) Demethylation activity of D135S versus the catalytically dead mutant from *E. coli* crude lysates on m^1^G in the RNA. Error bar indicates SD, n≥3.

Based on the high similarity of the core domain between Broccoli and Spinach (12), we depicted the potential Broccoli structure based on the crystal structure of Spinach (Figure 1B) (13). Due to the difficulty to chemically synthesize the full-length Broccoli RNA (51 nt) with an internal modification, we used a 33 nt Broccoli RNA segment in our assay (Figure 1A, B), as in Zhou *et al.* (7). In our design, the modified RNA segment is reverse transcribed using a specific RT that stops at the modification site, generating truncated cDNA product. If the modification is first removed by AlkB treatment, reverse transcription produces full-length cDNA product. In order to facilitate detection of fluorescence signal, the cDNA is amplified by PCR with primers that introduce a T7 promoter sequence, which enables *in vitro* transcription to amplify the amount of the full-length Broccoli RNA (Figure 1A). Broccoli RNA with internal modification that disrupts the folding of the aptamer has been previously used for measuring demethylation activity of FTO towards *N^6^*-methyladenosine (m^6^A) (14); however, the assay includes no amplification step thus requires a large amount of input RNA aptamer. In contrast, our strategy utilizes the property of the modification to block reverse transcription. The steps of cDNA amplification and *in vitro* transcription enable low amount of RNA input in our assay.

We evaluated our strategy using a known combination of demethylase and RNA modification: the AlkB mutant D135S and the m^1^G modification (Figure 1B, C). RNA containing m^1^G is a poor substrate for the wild type AlkB (15). AlkB D135S was previously identified as an efficient m^1^G demethylase by rational design (6). Before conducting the demethylation reaction, we evaluated the Broccoli RNA aptamer-based fluorescence system using pure RNAs (Supplementary Figure S1). The m^1^G-modified RNA produced background signals at low PCR cycles as expected; however, at high PCR cycles fluorescent RNA was also produced, suggesting that the M-MLV RT used in our study can read through the m^1^G site at low frequency. Nevertheless, the m^1^G-modified RNA produced much lower fluorescence signal than the unmodified RNA; the difference was ∼3.5-fold at 15 PCR cycles, confirming that the assay is capable of distinguishing modified and unmodified RNA. Furthermore, the fluorescent read-out can be fine-tuned with PCR cycle numbers to adjust the dynamic range and selection pressure of the assay (Supplementary Figure S1).

To facilitate high-throughput screening, it is critical to react our RNA substrate directly with AlkB expressing cell lysate, instead of purified protein. We envision several potential barriers to establish this assay. First, crude *E. coli* lysate may introduce a background signal stemming from the endogenous AlkB activity, other nucleic acids and cellular metabolites. Second, ribonucleases in the lysate may also cause a severe reduction of the signal. To control for these factors, we made a catalytically dead mutant of AlkB D135S by introducing alanine mutations at two critical positions of D133 and H131 (AlkB D135S D133A H131A) as a negative control and used RNase inhibitor in our assay. The AlkB D135S and the dead mutant were confirmed to express at the same level in *E. coli*, whereas the protein in the uninduced lanes may represent both endogenous AlkB and leaky expression of the expression plasmid (Figure 1D). By comparing the signal generated with AlkB D135S to that with the dead mutant, we could identify the effect solely derived from the demethylation activity of the overexpressed protein. Besides, the addition of RNase inhibitor decreased the potency and variations of RNase activities between samples (Supplementary Figure S2). Lastly, to enable high-throughput screening, this assay requires an RT that stops at the m^1^G site as much as possible and is cost-effective to allow screening at a large scale. We found that the commercial reverse transcriptase M-MLV could be used at low cost and stopped at m^1^G sites when dNTPs were used at 100 times lower concentration (5 μM final) than recommended by the manufacturer (Supplementary Figure S3). With these considerations, we applied the crude lysate of *E. coli* overexpressing AlkB mutants to the assay. As expected, the dead mutant produced low level of fluorescence, likely due to the endogenously expressed AlkB and the low-level read-through at the modification site by the reverse transcriptase. Notably, D135S produced ∼2.5-fold higher fluorescence than the dead mutant, demonstrating that our assay was able to detect the known m^1^G demethylation activity of AlkB D135S in crude lysate of *E. coli* with a high signal-to-noise ratio (Figure 1E). To achieve a high-throughput format, products from each step of reaction were used directly in the following step without purification.

### Screening at positions 135 and 210 of AlkB identified mutants with improved activity

**Figure 2.**
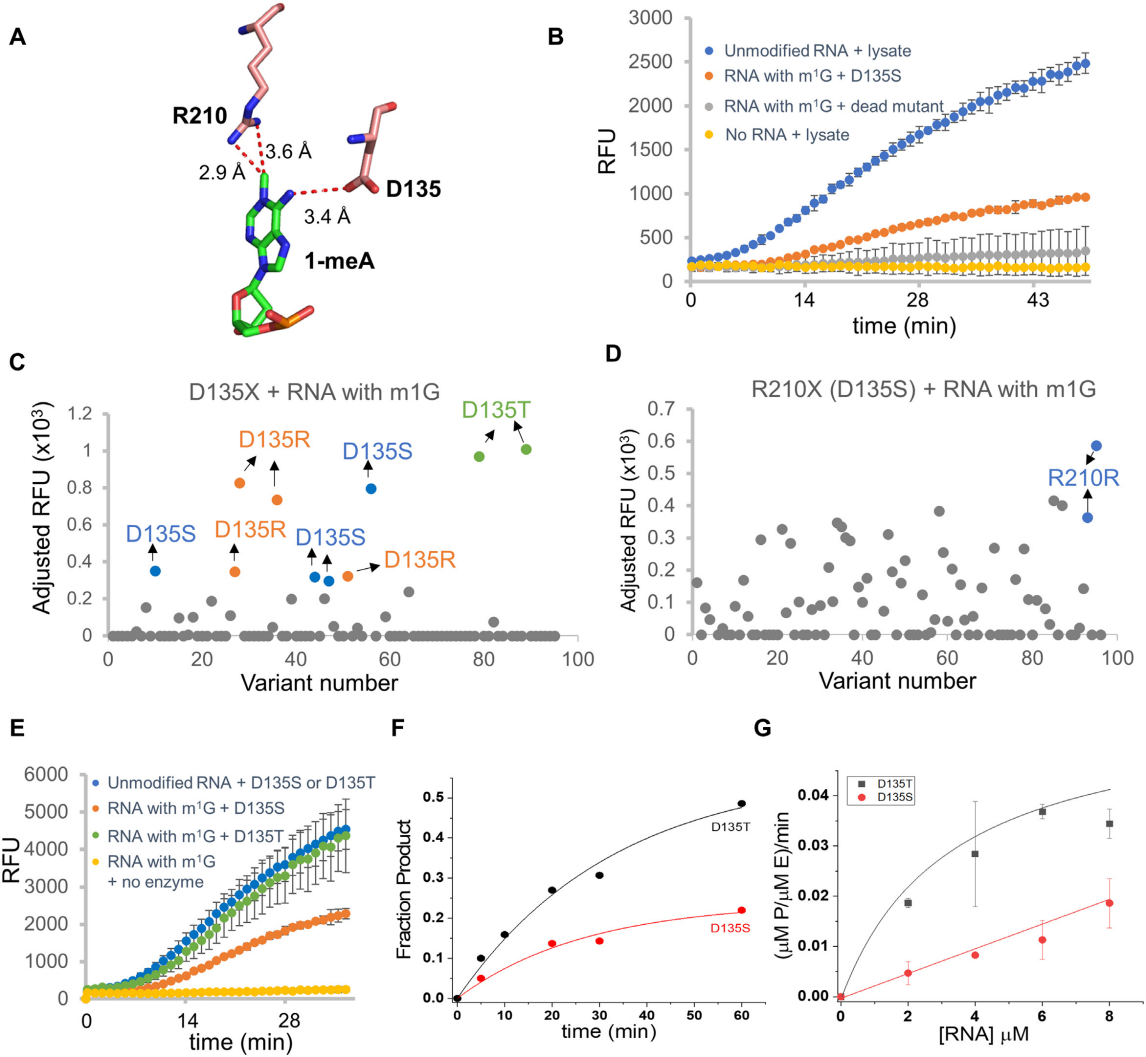
Screening at positions D135 and R210 of AlkB. (**A**) Residues D135 and R210 are located in close proximity to the modified nucleobase in the crystal structure (3BIE) (16). (**B**) Under the optimized condition, a high dynamic range was achieved to allow for the identification of mutants with higher reactivity. Error bar indicates SD, n≥3. (**C, D**) Screening results for libraries D135X and R210X (D135S). Each dot represents one variant in the library. The absolute fluorescence signal generated with each member in the library was adjusted by subtracting it from the signal generated with the dead mutant. Variants with adjusted signals below zero were presented as zero to indicate that no activity was observed for these variants under the condition. (**E**) *In vitro* demethylation assay with purified D135T and D135S mutants using the fluorescence assay confirmed that D135T has higher activity than D135S to demethylate m^1^G. The demethylation activity was judged by comparing with the signal of the positive control, which was the unmodified RNA incubated with the respective variant. For direct comparison between the two variants, data were adjusted so that the positive controls for the two variants had the same level of fluorescence signals. (**F**) Plot of fraction of product as a function of time for the reaction containing 4 μM enzyme and 4 μM 9-mer m^1^G-RNA. (**G**) Plot of (μM P/μM E)/min as a function of RNA concentration for reactions containing 4 μM enzyme. The value of (μM P/μM E)/min was calculated as ([S]/[E])*A*k (see Materials and Methods). Data shown are the average and standard deviation of three independent reactions for each condition.

Next, we applied the assay utilizing the m^1^G-broccoli RNA to screen for variants in functionally important residues in AlkB. According to the crystal structure of the wild type AlkB bound to a dsDNA substrate bearing an m^1^A modification, residues D135 and R210 in the active site are located in close proximity to the N6 and the N1 position of the target nucleobase, respectively (Figure 2A). The ionic interaction between D135 and the N6 position suggests that variations at D135 may accommodate nucleobases that differ from m^1^A at the N6 position. Indeed, our laboratory has found that D135S mutation possesses higher demethylation activity towards m^1^G than the wild type enzyme, and the Yi laboratory has found that the D135I mutation enables the enzyme to demethylate m^6^A in RNA at ∼1/3rd activity of the natural m^6^A demethylation enzyme ALKBH5 (6,10). The side chain of R210 is within van der Waals contact with the methyl group of the nucleobase m^1^A, suggesting an important role in the recognition of N1 methylation. R210 is in a characteristic RXXXXXR motif of the AlkB family proteins and is also thought to play a role in anchoring the co-substrate α-ketoglutarate in place through salt bridging interactions (2,16). We chose to systematically evaluate these two residues in order to gain more insights into the structure-activity relationship as well as to identify potential mutants with higher enzymatic activities towards m^1^G.

In order for the assay to distinguish various levels of demethylation activities, we optimized the assay condition to allow for a large dynamic range. We tested several parameters including the reaction time, the amount of crude lysate used, the RNA substrate input and PCR cycle numbers which collectively affect the sensitivity and dynamic range of the assay (Supplementary Figure S4). Aiming to identify AlkB mutants with improved activity beyond D135S, we settled on a condition where the fluorescence signal generated with D135S reached 40% of that of the wild-type Broccoli sequence, but remained distinct from the signal generated with the dead mutant at the final time point of reaction of around 50 min (Figure 2B). This condition allows for a large dynamic range to identify variants with higher activity.

With the optimized condition, we conducted screening studies for positions D135 and R210 of AlkB. Saturation mutagenesis was performed to generate the D135X library and the R210X library. Since wild type AlkB does not demethylate m^1^G, we also introduced the D135S mutation in addition to the mutation introduced at position 210 to make the R210X (D135S) library. Each library was screened in a 96-well plate, ensuring coverage of the theoretical library diversity. The genotypes of mutants with various levels of fluorescence signals (adjusted relative fluorescent unit or RFU) are summarized in Supplementary Table S1. Importantly, we found that D135S generated higher adjusted RFU values than the wild type AlkB enzyme, in agreement with D135S having a known higher activity than the wild type protein towards m^1^G and confirming the capability of the assay to distinguish different levels of demethylation activities (Figure 2C). D135R and D135S generated similar adjusted RFU on average, suggesting that the mutant D135R is as active as mutant D135S in m^1^G demethylation (Figure 2C and Supplementary Table S1). Interestingly, mutant D135T had ∼2-fold higher adjusted RFU value than D135S, suggesting that D135T is more active than D135S (Figure 2C and Supplementary Table S1). To further test this observation, we purified D135T and D135S enzymes to > 90% purity and evaluated their activities using the m^1^G-broccoli fluorescence assay. Consistent with the screening, D135T generated ∼2-fold higher fluorescence signals than D135S (Figure 2E). To more directly compare catalytic efficiencies of these two mutants, we used a 9-mer RNA oligo containing an m^1^G modification as the substrate and measured demethylation activities under different substrate concentrations with MALDI-TOF/TOF MS. Our data showed that D135T (*k*_cat_/*K*_M_ = 15.7 M^−1^min^−1^) exhibited around 7-fold higher catalytic efficiency than D135S (*k*_cat_/*K*_M_ = 2.2 M^−1^min^−1^) (Figure 2F, G, Table 1).

**Table 1.** Kinetic constants of D135T and D135S demethylating m^1^G in the RNA oligo 5′-GAGC(m^1^G)UUAG

Enzyme	*k*_cat_ (min^−1^)	*K*_M_ (μM)	*k*_cat_/*K*_M_ (M^−1^min^−1^)
D135T	0.052 ± 0.008	3.3 ± 1.3	15.7 ± 3.7
D135S	-	-	2.2

The screening at position R210 failed to identify mutants with higher activities than the original R210 (D135S) (Figure 2D). Nevertheless, our data showed that some amino acids at this location, including Gly, Ser, Pro, Asp and Leu, could function similarly well as the wild type amino acid Arg, judging from the fact that these mutations had adjusted RFU values of 57–70% of that of the parent protein (Supplementary Table S1). The accommodation of smaller amino acids here suggests that this residue may not be essential for the catalytic reaction nor the binding to the ligand αKG.

### D135T and D135S facilitate tRNA sequencing experiments with comparable efficiency

**Figure 3.**
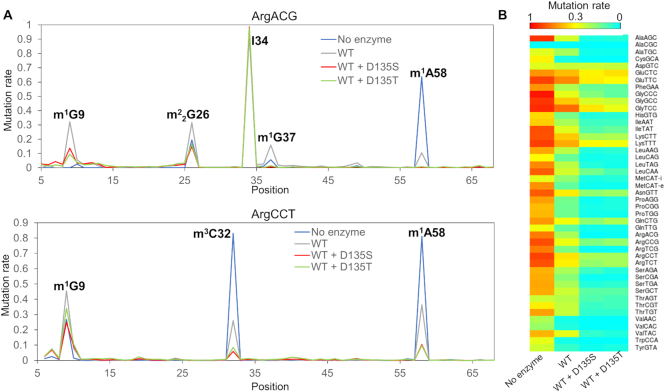
D135T exhibited similar capacity as D135S to remove human tRNA modifications in tRNA-seq experiments. (**A**) Mutation rate in sequencing of HEK293T samples for tRNA^Arg^(ACG) and tRNA^Arg^(CCT) in samples of no enzyme, wild type AlkB (WT), wild type AlkB plus the D135S mutant, and wild type AlkB plus the D135T mutant. I34 has an A-to-G mutation signature under all circumstances and is not sensitive to AlkB treatment. (**B**) Heatmap showing mutation rates at m^1^A58 for all cytosolic tRNAs under the four conditions in tRNA-seq. Mutation rates are shown for the highest expressed tRNA isodecoder among each isoacceptor family (27).

The AlkB mutant D135S has been used in tRNA-seq experiments (6) because of its considerably higher enzymatic activity than the wild type protein to remove tRNA modifications. Since we identified D135T having a higher activity than the D135S mutant for m^1^G in our system, we applied D135T in tRNA-seq experiments. We first tested D135S and D135T in removing modifications from total yeast tRNA through LC-MS/MS. Under our condition (see Methods), both D135T and D135S efficiently removed > 90% of m^1^A (located in the T loop), 40–60% of m^1^G (located in both anticodon loop and position 9 in the tRNA tertiary structure) and around 50% of m^3^C from yeast tRNA (Supplementary Figure S5). We observed that D135T exhibited a marked trend of slightly higher efficiency to remove the m^1^G and the m^3^C modifications (Supplementary Figure S5). The less prominent difference in the observed activities between D135T and D135S was probably due to the tRNA substrate used and the high enzyme to substrate ratio (2.5:1) employed in this particular experiment. We then compared tRNA-seq results using total RNA from HEK293T cells treated with D135T in combination with wild type AlkB, D135S in combination with wild type AlkB, wild type AlkB alone, or no enzyme. We used the same demethylation conditions as in the published tRNA-seq work (6) which included 4-fold molar excess of AlkB enzymes over tRNA substrate. Our tRNA-seq results showed that D135T and D135S remove methylations similarly at five main locations in the human tRNA (Figure 3 and Supplementary Figure S6). Specifically, both enzymes in combination with the wild type protein efficiently removed m^1^A58, m^1^G37 and m^3^C32 located in loop regions in tRNA and moderately removed m^1^G9 and m^2^_2_G26 in structured regions in tRNA (Figure 3 and Supplementary Figure S6). Divergent results were also observed, such as D135S exhibited slightly higher efficiency than D135T in removing some sites such as m^1^A58 in tRNA^Glu^(CTC) and m^1^G37 in tRNA^Leu^(CAA), whereas D135T exhibited better results for sites including m^1^A58 in tRNA^Gly^(TCC), and m^1^G9 in tRNA^Arg^(CCG) (Figure 3 and Supplementary Figure S6).

### Expanding th**e screening platform towards methylation damage in DNA**

The *E. coli* AlkB has long been known as a DNA repair enzyme which can catalyze the repair of m^1^A and m^3^C in single stranded DNA (1). New functionalities of AlkB towards other DNA damages would contribute to the DNA repair toolbox. For example, O6-methylguanosine (O6mG) is a severe DNA damage that causes G to A mutation during genome amplification (17–19). Earlier studies have demonstrated that this damage is among the most carcinogenic alkylated bases (20). The O6mG repair is carried out by O6-methylguanine-DNA methyltransferase (MGMT in human and Ada in *E. coli*); however, the active form of MGMT cannot be regenerated after one round of catalysis, limiting its repair capacity especially in the case of excessive damage levels (21,22). We envision an engineered AlkB as an exogenous source to repair O6mG in DNA.

**Figure 4.**
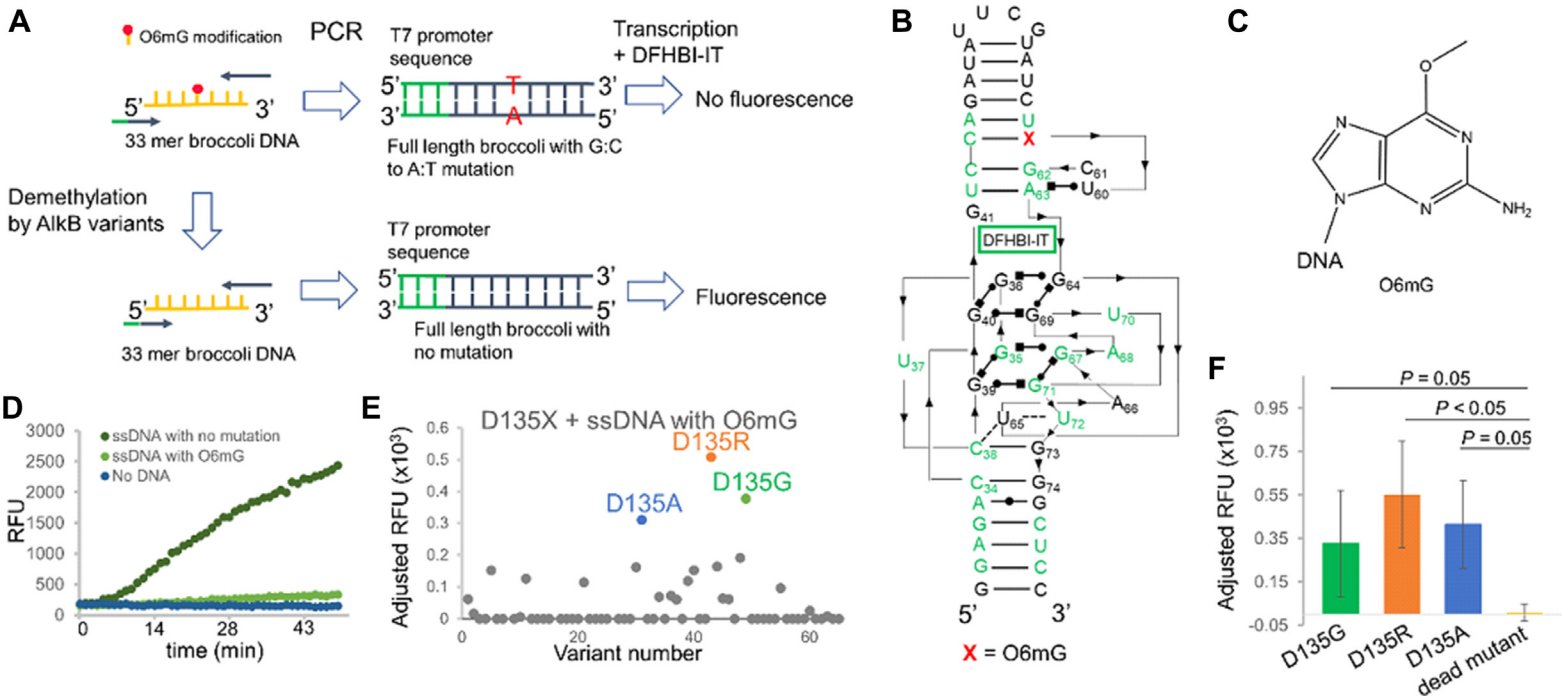
Application of the screening assay to a DNA substrate. (**A**) The scheme of the Broccoli RNA fluorescence assay for high-throughput screening of AlkB activity towards O6mG in DNA. (**B**) The Broccoli DNA substrate with O6mG used in the assay. Conserved regions are labeled in green. O6mG is located at site 59. (**C**) The chemical structure of O6mG. (**D**) ssDNA with O6mG produced much lower level of fluorescence than the unmodified DNA. (**E**) Screening of the D135X library against O6mG identified positive hits. The absolute fluorescence signal generated with each member in the library was adjusted by subtracting it from the signal generated with the dead mutant. Variants with adjusted signals below zero were presented as zero to indicate that no activity was observed for these variants under the condition. (**F**) Reproducing positive signals of hits from the screening using the fluorescence assay. Error bar indicates SD, *n* ≥ 3. Statistical differences between the positive hits and the dead mutant were determined by Welch's *t* tests.

We modified our assay in several ways to apply it to the DNA system: (i) a 51 mer DNA Broccoli sequence bearing an O6mG modification at a specific G position was used as the substrate; (ii) following the demethylation reaction, the DNA substrate was directly used in PCR followed by an *in vitro* transcription, thus omitting the RT reaction; (iii) an error-prone polymerase (Taq polymerase) was used in the PCR reaction to introduce a G-to-A mutation at the modification site (Figure 4A–C). Presumably, the DNA substrate containing O6mG will generate a Broccoli RNA with a G-to-A mutation more often than an unmodified substrate. Mutated product does not fold properly, thus generating diminished fluorescent signal.

We screened four G positions (G_41_, G_46_, G_53_ and G_59_) in the Broccoli sequence and found that G_59_ is highly sensitive to the G-to-A mutation (Supplementary Figure S7). Mutating the G to an A at this position led to a dramatic decrease in the fluorescence signal (Supplementary Figure S7). We thus used a chemically synthesized 51 nucleotide Broccoli DNA harboring O6mG at this location in our assay. We confirmed that the DNA with the O6mG modification generated a much lower fluorescence signal compared to the unmodified DNA (Figure 4D). We chose to use single-stranded DNA for our screening, as AlkB is known to prefer ssDNA substrates and O6mG can be found in single-stranded regions of DNA associated with replication forks (18,23).

According to the crystal structure, one residue in AlkB that makes contacts to the O6 position of the G modification is again D135; thus, we screened the D135X library for demethylating the ssDNA substrate harboring the O6mG modification. We identified several positive hits from the screening, including D135R, D135G and D135A (Figure 4E). We further confirmed these three positive hits using the fluorescence assay with crude *E. coli* lysate expressing these variants and were able to reproduce the results (Figure 4F). Further evaluations will be needed to confirm the demethylation activity of these mutants. These results demonstrate the applicability of our system to screen for AlkB mutants with activity against modifications in DNA substrates and can serve as a starting point for further refinement of an AlkB-derived O6mG demethylase.

## DISCUSSION

*Escherichia coli* AlkB has the potential to be engineered for new functionalities (7,8,10). Here, we introduce a high-throughput screening assay which can substantially accelerate identification of AlkB variants with new activities toward unnatural substrates such as m^1^G in RNA or O6mG in DNA. We have demonstrated that demethylation is possible with crude lysate of *E. coli* expressing AlkB variants, eliminating several purification steps and facilitating high throughput application. Several factors can affect the sensitivity and dynamic range of the assay, including lysate and substrate input, reaction time, and PCR cycle numbers. These parameters may need to be optimized empirically to accommodate the study of other types of nucleic acid modifications and the types of AlkB variant libraries.

Here, we have shown two applications of our assay. By conducting screening of variants for positions D135 and R210, we were able to identify mutants with similar or even higher activity than the endogenous enzyme, corroborating the quantitative capacity of the assay. According to the sequence alignment of natural *E. coli* AlkB and its homologs surrounding D135 and R210, some species have a serine at position corresponding to 135 (Supplementary Figure S8); this is compatible with our results here showing that D135S possesses high catalytic efficiency. Although R210 is in the relatively conserved RXXXXXR motif, our data showing that R210 can be replaced with other amino acids without substantially decreasing the activity of the enzyme suggests that this residue may not be essential for the activity of the protein. We also used mutants D135T and D135S identified here in tRNA-seq experiments. Results from the tRNA-seq experiments indicate that these two mutants may be used jointly to achieve maximum removal of methylations in tRNA-seq experiments. D135R was also found to be a hyperactive mutant towards m^1^G; it may also be useful in tRNA-seq experiments. One future test of using these AlkB variants is to vary the amount of each enzyme in the sequencing reaction to obtain a better assessment on their usefulness. We have also applied our assay to the O6mG damage in a DNA substrate. Our studies revealed several candidate AlkB variants conferring weak activity towards this damage. Other mutations to further improve the reactivity towards repairing O6mG are to be explored.

We noticed that there was variability in fluorescence readout for the same variant in the 96-well screening (Figure 2C, D). This was likely due to the difference in the cell growth and lysate preparation between different wells. In an evaluation test of D135T in a 96 well plate, we found that the adjusted RFU values ranged from 453 to 1427 (*n* = 12), with an average adjusted RFU value of 800, consistent with the screening result for the D135X library (Figure 2C and Supplementary Table S1). This suggests that the readout can be variable and further optimization should be done to increase the accuracy and decrease the false negative rates. For examples, the variation in cell growth and lysate preparation can be mitigated by measuring cell density to ensure the same number of cells in the seeding and lysis steps. It would also be helpful to perform an SDS-PAGE analysis to confirm protein expression before further proceeding. Increasing the number of colonies in the screening can also help increase the accuracy. In addition, further validation and biochemical characterization of purified proteins are needed to test the candidates from the screening, as we have done here for D135T.

We envision several other immediate applications of our screening system. First, the assay can be used to study other important residues in AlkB or other demethylases against various types of modifications (Supplementary Figure S8B-C). Although the D135S and D135T, together with the previously reported D135S/L118V, possess higher reactivities than the wild type AlkB to remove m^1^G and m^2^_2_G, additional mutants with better activity will still be desired for RNA-seq applications. Comprehensive screenings at position L118 and other positions proximal to N2 of guanosine such as M61 may help identify better variants towards m^2^_2_G (Supplementary Figure S8B and C). In addition, selection for proteins with robust activity at higher reaction temperatures may facilitate removal of modifications that are otherwise buried in the tRNA tertiary structure such as m^1^G9. Second, it is desirable to screen for AlkB mutants with better specificity. Examples include mutants capable of more stringent discrimination between m^1^G and m^2^_2_G to facilitate the identification of either modifications in the mRNA transcriptome. Third, the assay can be used to screen for small molecule inhibitors of AlkB and homologs. Human ALKBH2 and ALKBH3 are involved in diseases and studies identifying selective inhibitors are of therapeutic potential (24). Lastly, as substrates of AlkB and homologs have not been completely revealed and substrate specificity not fully understood (2,25), our screening system may facilitate biochemical studies for these intriguing enzymes.

## DATA AVAILABILITY

RNA-seq data has been deposited to GEO under accession number: GSE152694. The scripts for data analysis have been deposited in GitHub: https://github.com/yuruwang26/tRNA_seq_scripts.

## Supplementary Material

gkaa1213_Supplemental_File
