# Supplementary material for: A high-throughput screening method for evolving a demethylase enzyme with improved and new functionalities
Source: Nucleic Acids Res. 2020 Dec 18;49(5):e30. doi: 10.1093/nar/gkaa1213 (PMC7968990; doi:10.1093/nar/gkaa1213)
Supplement: gkaa1213_Supplemental_File [file gkaa1213_supplemental_file.pdf]

Supporting Information

A High-throughput Screening Method for Evolving a Demethylase Enzyme with Improved and New Functionalities

Yuru Wang[1,2], Christopher D. Katanski[1], Christopher Watkins[1], Jessica N. Pan[1], Qing Dai[2], Zhuoxun Jiang[1], Tao Pan[1,]*

1. Department of Biochemistry and Molecular Biology, 2. Department of Chemistry, University of Chicago, Chicago, IL 60637, USA

*corresponding author: taopan@uchicago.edu

Contents

Figure S1. Evaluating the broccoli RNA fluorescence assay using pure synthetic RNAs 2

Figure S2. Testing the effect of RNase inhibitor on the RNase activity in the *E.coli* crude lysate using the RNase Activity Detection/Quantification Assay Kit..3

Figure S3. Testing the M-MLV RT in reverse transcription with the m^1G-broccoli RNA as the template..3

Figure S4. Optimizing the condition for screening experiments 4

Figure S5. LC-MS/MS to measure the demethylation efficiency of D135T and D135S on yeast tRNA 4

Figure S6. Heatmap showing mutation rates at m^1G9, m^1G37, m^2_2G26 and m^3C32 for cytosolic tRNAs under the four conditions in tRNA-seq...5

Figure S7. Identifying guanosine residues in the broccoli sequence that are sensitive to the G-to-A mutation 6

Figure S8. The sequence alignment of AlkB family proteins and candidate residues for screening studies. 7

Table S1. The summary of screening results for residues D135 and R210 of AlkB 8

Table S2. RNA and DNA oligonucleotides (5’ to 3’) used in this study..8

Reference 9

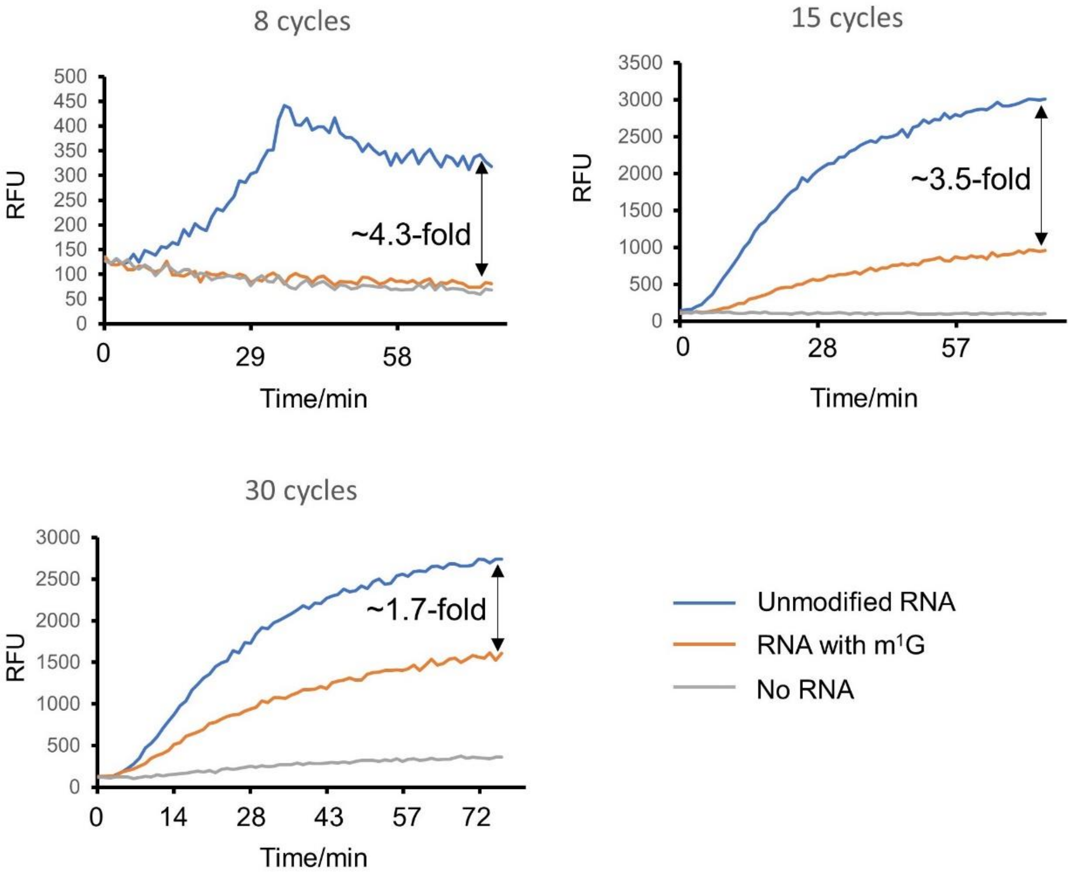

Figure S1. Evaluating the Broccoli RNA fluorescence assay using pure synthetic RNAs. The assay was tested with different numbers of cycles in the PCR reaction.

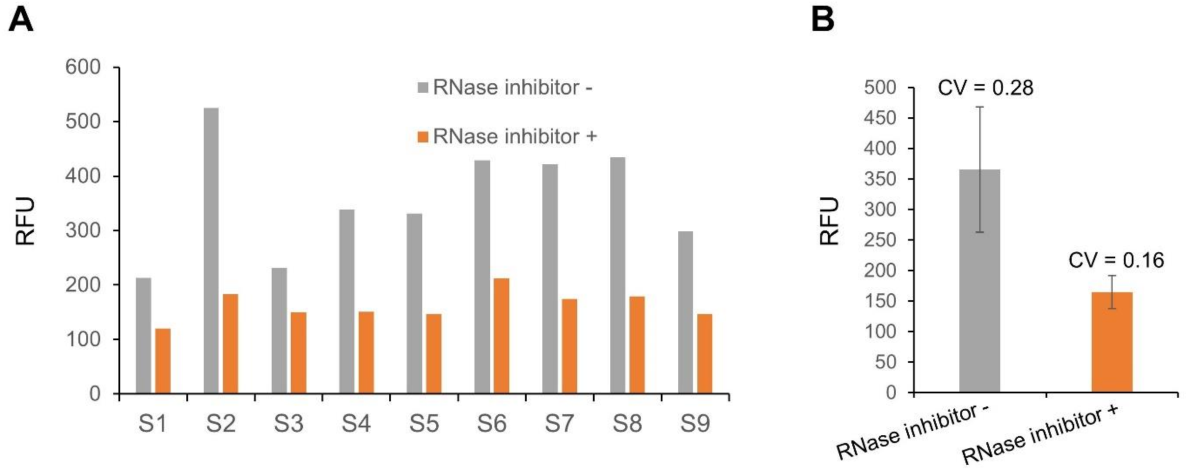

Figure S2. Testing the effect of RNase inhibitor on the RNase activity in the *E. coli* crude lysate using the RNase Activity Detection/Quantification Assay Kit. (A) Fluorescence (whose relative intensity corresponds to relative RNase activity) of biological replicates 1 to 9 (S1-S9) with or without the addition of RNase inhibitor. (B) Summarization of data in (A) with statistics shown. Error bars mean SD, N=9. CV means coefficient of variation.

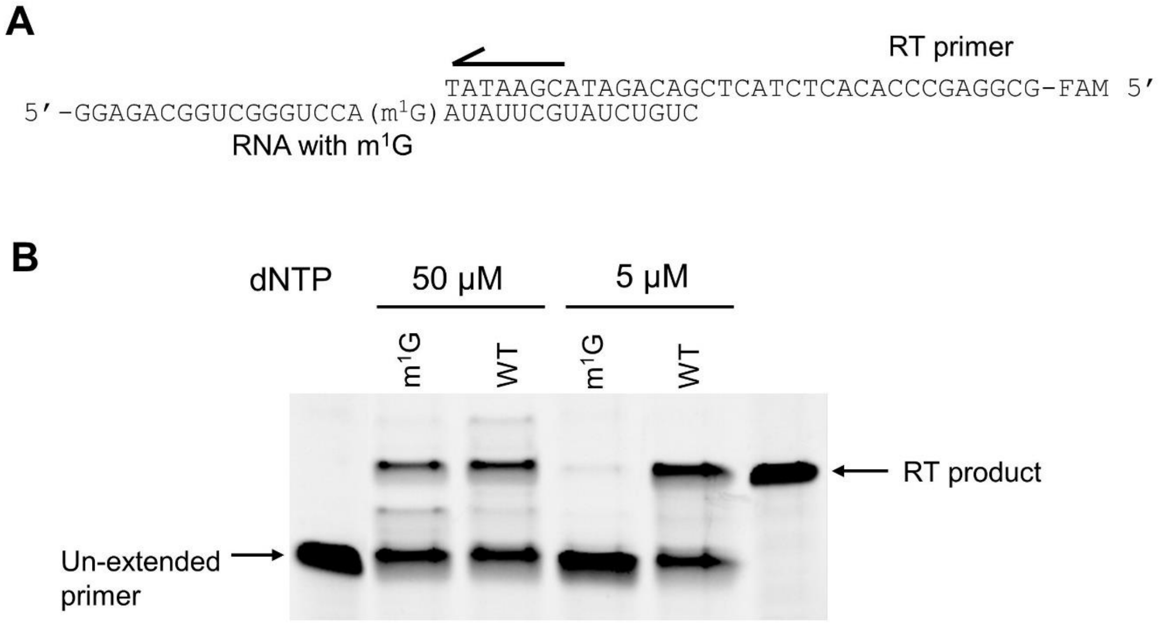

Figure S3. Testing the M-MLV RT in reverse transcription with the m^1G-broccoli RNA as the template. (A) Sequences of the RNA substrate and the RT primer used in the assay. (B) Testing the primer extension with dNTP at 10 times lower (50 μM final) and 100 times lower (5 μM final) concentrations than recommended by the manufacturer. WT means unmodified RNA.

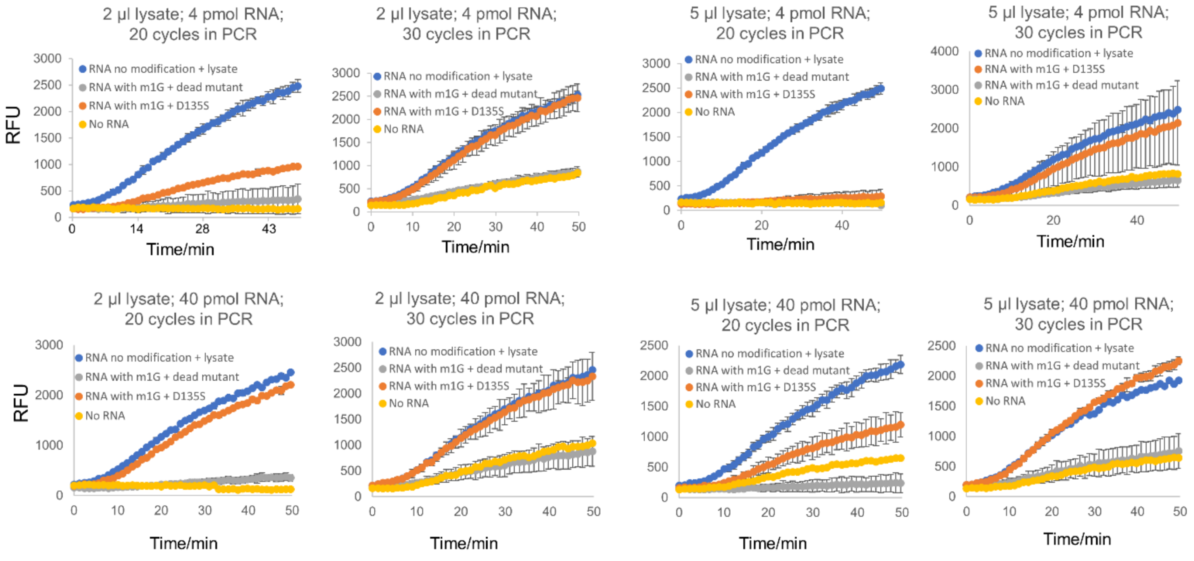

Figure S4. Optimizing the condition for screening experiments. The parameters tested include the amount of lysate, the amount of RNA and cycle numbers in PCR. The condition of 2 µl lysate, 4 pmol RNA and 20 cycles in PCR was used in the screening experiments for positions D135 and R210 using the m^1G-broccoli RNA as the substrate. Error bar indicates SD, n≥3.

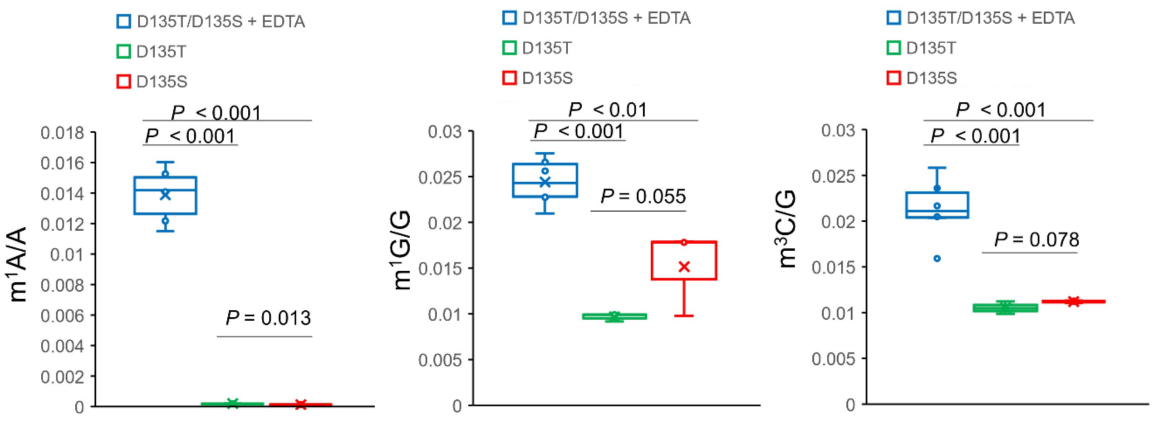

Figure S5. LC-MS/MS to measure the demethylation efficiency of D135T and D135S on yeast tRNA. Data for m^1A and m^1G were obtained after 10 min of demethylation reaction; data for m^3C was obtained after 30 min of demethylation reaction. Error bar indicates SD, n≥3.

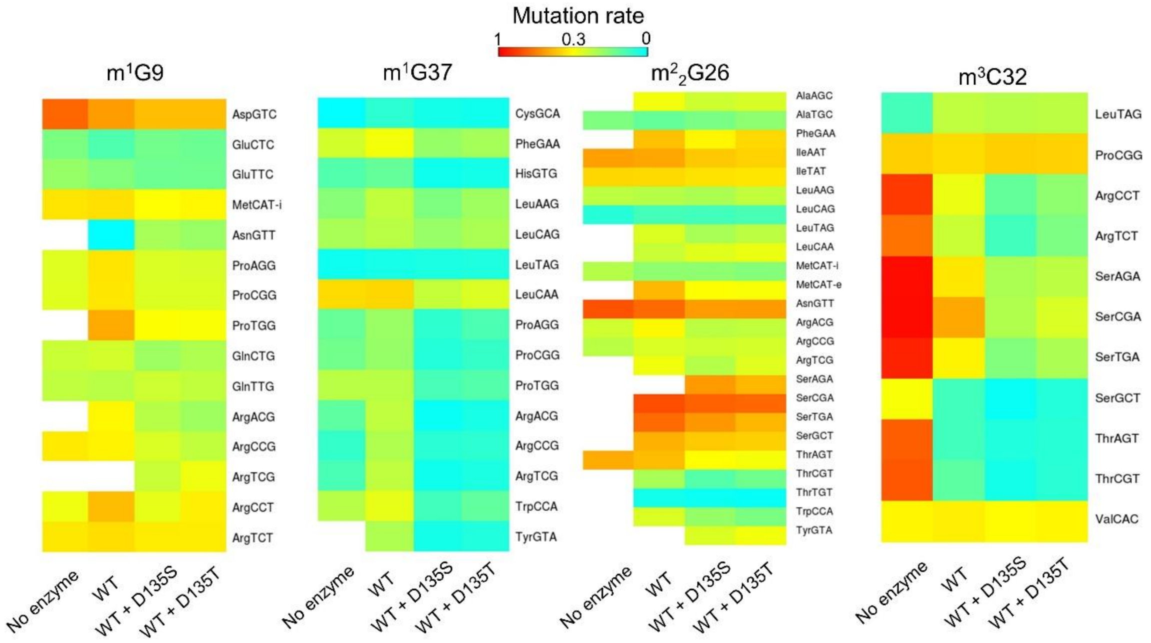

Figure S6. Heatmap showing mutation rates at m^1G9, m^1G37, m^2_2G26 and m^3C32 for cytosolic tRNAs under the four conditions in tRNA-seq. The tRNA samples were treated with no enzyme, wild type AlkB (WT), wild type AlkB plus the D135S mutant (WT + D135S) or wild type AlkB plus the D135T mutant (WT + D135T).

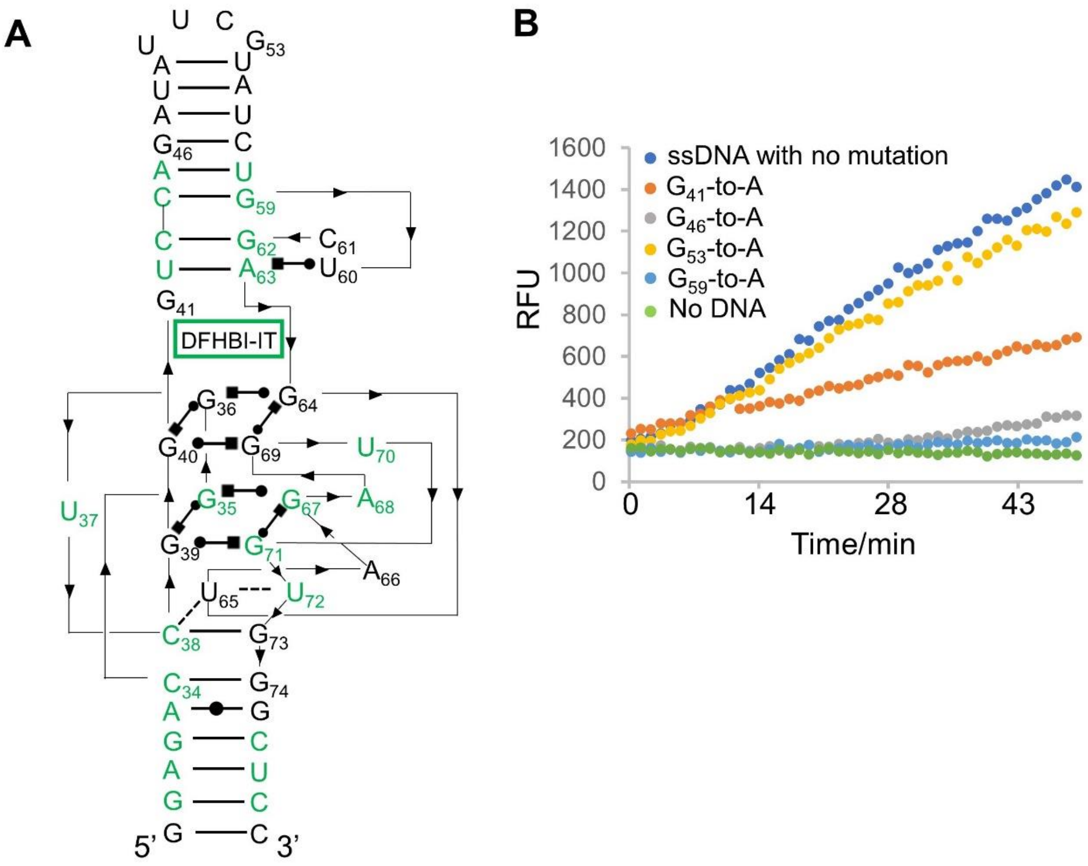

Figure S7. Identifying guanosine residues in the broccoli sequence that are sensitive to the G-to-A mutation. (A) The DNA sequence of the broccoli aptamer with conservative bases labeled in green. (B) The fluorescence signal generated from the broccoli sequence with the guanosine located at site 41, site 46, site 53 or site 59 mutated to an adenosine. Site 59 showed the highest sensitivity upon the G-to-A mutation. The numbering scheme of the Broccoli RNA is based on the ref (1).

A

```
                                D135                                          R210
E. coli AlkB             130   LHQDKDEPDLRAPIV-SVSLGL… …LTIDCRYNLTFRQAGKKE-----  216
D. Melanogaster Alkb     203   GHTDHSEPNKSAPLF-SFSFGQ… …YMDDSRININIRQVLNPGDVKL-  332
S. pombe abh1            204   AHIDESEEDLTLPLI-SLSMGL… …WISRKRVNFNVRQV-RPSR----  302
C. elegans alkb-1        244   PHVDKSE-RSAAPLI-SMSLGQ… …YLNSSRVNFTIRQV-NPN-----  359
H. sapiens ALKBH2        169   GEHRDDERELAPGSPIASVSFG… …KVLAPRVNLTFRKILLTKK----  261
H. sapiens ALKBH3        189   DWHSDDEPSLGRCPIIASLSFG… …HSREPRVNLTFRTVYPDPRGAPW  286
M. musculus Alkbh3       189   DWHSDDEPSLGSCPVIASLSFG… …HSRQPRVNLTFRTVYPDPRGAPR  286
```

B

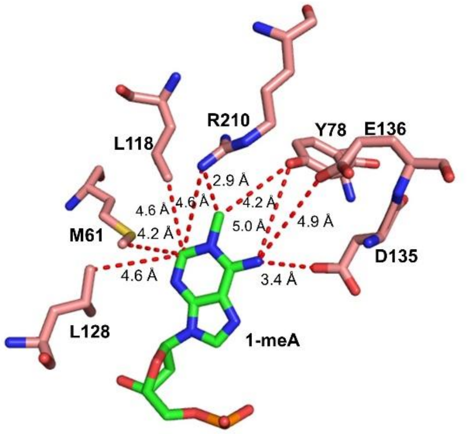

C

Proximal to	Residues	Target modifications
N6	Y78, D135, E136	m^1G, m^6A
N1	R210, Y78	m^1A, m^1G
C2	M61, L118, L128, R210	m^2G, $m^2{}_2G$

Figure S8. The sequence alignment of AlkB family proteins and candidate residues for future screening studies. (A) In the sequence alignment, residues aligned to D135 in *E. coli* AlkB are highlighted in cyan (D) and magenta (S). Residues aligned to R210 in *E. coli* AlkB are highlighted in yellow. The sequence alignment was made using the NCBI blast tool. (B) Residues in *E. coli* AlkB that make ionic interactions or are within van der Waals contacts with the modified nucleobase m^1A in a dsDNA substrate (3BIE) (2). (C) A summary of candidate residues that may be screened to identify variants with improved activities towards different modifications.

Activity pool \ library	D135X	R210X (D135S)
I	T (2, 0.99)	R (2, 0.47)
II	R (3, 0.49), S (5, 0.48), I (1, 0.2)	G (5, 0.32), S (2, 0.29), P (2, 0.33), D (1, 0.267), L (1, 0.29)
III	D (WT, 3, 0), A(2, 0), G(1, 0), V(1, 0), L(1, 0), M(1, 0), C(1, 0)	T (1, 0), A (1, 0), Q (1, 0)

Table S1. The summary of screening results for residues D135 and R210 of *E. coli* AlkB. The first number in the parenthesis is the number of times the amino acid was identified in the screening; the second number in the parenthesis is the average value of adjusted RFU (x10^3) for the variant.

Table S2. RNA and DNA oligonucleotides (5' to 3') used in this study.

RNA with m1G	GGAGACGGUCGGGUCCA(m^1G)AUAUUCGUAUCUGUC
9-mer RNA oligonucleotide	GAGC(m^1G)UUAG
Unmodified RNA	GGAGACGGUCGGGUCCAGAUAUUCGUAUCUGUC
ssDNA with O6mG	GCGG AGA CGG TCG GGT CCA GAT ATT CGT ATC T(O6mG)T CGA GTA GAG TGT GG GCT
ssDNA with no mutation	GCGG AGA CGG TCG GGT CCA GAT ATT CGT ATC TGT CGA GTA GAG TGT GG GCT
RT primer	GCGGAGCCCACACTCTACTCGACAGATACGAATAT
RT primer (FAM)	5' FAM- GCGGAGCCCACACTCTACTCGACAGATACGAATAT
RT-PCR-for	CTAATACGACTCACTATAGGGCGGAGACGGTCGGG
RT-PCR-rev	GCGGAGCCCACACTCTACTCGACAGATACGAATAT
Broccoli truncation	5' FAM- GCGGAGCCCACACTCTACTCGACAGATACGAATATCTGGA
Broccoli full-length	5' FAM- GCGGAGCCCACACTCTACTCGACAGATACGAATATCTGGACCCGACCGTCTCCG
D133A-H131A-for	GCGCGAAACTGTCGCTGGCTCAGGCGAAATCTGAACCGGATCTGCGCGC
D133A-H131A-rev	GCGCGCAGATCCGGTTCAGATTTCGCCTGAGCCAGCGACAGTTTCGCGC
D135X-for	CTGTCGCTGCATCAGGATAAANNSGAACCGGATCTGCGCGCGCCAATTG
D135X-rev	CAATTGGCGCGCGCAGATCCGGTTCSNNTTTATCCTGATGCAGCGACAG
R210X-for	CCGCTACAACCTGACATTCNNSCAGGCAGGTAAAAAAGAATAAC
R210X-rev	GTTATTCTTTTTTACCTGCCTGSNNGAATGTCAGGTTGTAGCGG

13-G-to-A	GCGGAGACGGTCGGATCCAGATATTCGTATCTGTCGAGTAGAGT GTGGGCTCCGC
18-G-to-A	GCGGAGACGGTCGGGTCCAAATATTCGTATCTGTCGAGTAGAGT GTGGGCTCCGC
25-G-to-A	GCGGAGACGGTCGGGTCCAGATATTCATATCTGTCGAGTAGAGT GTGGGCTCCGC
31-G-to-A	GCGGAGACGGTCGGGTCCAGATATTCGTATCTATCGAGTAGAGT GTGGGCTCCGC
DNA-screening-PCR-for	CTAATACGACTCACTATAGGGCGGAGACGGTCGG
DNA-screening-PCR-rev	GCGGAGCCCACACTCTACTCG

Reference

1. Filonov, G.S., Moon, J.D., Svensen, N. and Jaffrey, S.R. (2014) Broccoli: rapid selection of an RNA mimic of green fluorescent protein by fluorescence-based selection and directed evolution. *J. Am. Chem. Soc.*, **136**, 16299-16308.
2. Yang, C.G., Yi, C., Duguid, E.M., Sullivan, C.T., Jian, X., Rice, P.A. and He, C. (2008) Crystal structures of DNA/RNA repair enzymes AlkB and ABH2 bound to dsDNA. *Nature*, **452**, 961-965.
